# The Correlation between Complete Blood Count Parameters and Appendix Diameter for the Diagnosis of Acute Appendicitis

**DOI:** 10.3390/healthcare8010039

**Published:** 2020-02-13

**Authors:** Emin Daldal, Hasan Dagmura

**Affiliations:** 1 General Surgery, Gaziosmanpasa University, Kaleardı mahallesi, 60250 Tokat, Turkey; emin.daldal@gop.edu.tr; 2 General Surgery and Surgical Oncology Department, Gaziosmanpasa University, Kaleardı Mahallesi, 60250 Tokat, Turkey

**Keywords:** acute appendicitis, CRP, diameter, NLR

## Abstract

Acute appendicitis is one of the most common causes of acute abdominal diseases seen between the ages of 10 and 19, mostly seen in males. The lifetime risk of developing acute appendicitis is 8.6% for males and 6.7% for females. We aimed to investigate the efficacy of the complete blood count parameters, C-reactive protein, and Lymphocyte-C-reactive Protein Ratio laboratory tests in the diagnosis of acute appendicitis, as well as their relationship with appendix diameter. We retrospectively examined all patients who underwent appendectomy between 1 January 2012 and 30 June 2019 in the General Surgery Clinic of Gaziosmanpasa University Faculty of Medicine. Laboratory tests, imaging findings, age, and gender were recorded. Lymphoid hyperplasia is considered as normal appendix—in other words, as negative appendicitis. The distribution of Lymphoid hyperplasia and appendicitis rates were statistically different in the groups formed according to appendix diameter (≤6 and >6 mm) (*p* < 0.001). We found a significant correlation between appendix diameter and WBC (White blood count), Lymphocyte, Neutrophil, RDW(Red blood cell distribution width), NLR(Neutrophil to lymphocyte ratio), and PLT/L (Platelet to lymphocyte ratio), MPV (Mean platelet volume) and RDW were significantly different in patients with an appendix diameter of ≤6 mm (*p* = 0.007, *p* = 0.006, respectively). WBC, Neutrophil, PDW, and NLR values were significantly different between appendicitis and hyperplasia groups in patients with an appendix diameter of >6 mm. The sensitivity of the NLR score (cutoff = 2.6057) in the diagnosis of appendicitis was 86.1% and selectivity was 50% in these patients. Complete blood count parameters evaluation with the clinical findings revealed that NLR is an important parameter that may help the diagnosis of acute appendicitis with an appendix diameter of >6 mm. In patients whose pathological results indicated acute appendicitis but who had a diameter of ≤6 mm, we found an elevated MPV and low RDW values.

## 1. Introduction

Acute appendicitis (AA) is one of the most common acute abdominal diseases among patients presenting with abdominal pain. However, diagnosis may not always be possible [1,2,3,4]. Factors held accountable for AA are occlusion of the lumen, the development of mucosal ischemia after intraluminal pressure increase, and the addition of bacterial infection [1]. AA is most commonly seen between 10 and 19 years of age. Males have a higher rate in all age groups than females. The lifetime risk of developing AA is 8.6% for males and 6.7% for females [5,6]. The first appendectomy was performed by A. Grooves and, following R. Fitz’s article on perforated appendicitis in 1886, appendectomy became an effective treatment method [7]. Appendectomy is the preferred treatment modality in the treatment of AA. However, antibiotic treatment may also be considered in selected uncomplicated cases [3,7,8,9].

Although AA is one of the most common acute abdominal diseases, the correct diagnosis has been controversial. The negative appendectomy rate is about 15%. In women, this rate goes up to 26% [10]. Classic appendicitis presents with pain starting from the periumbilical region, anorexia, nausea, and vomiting. Approximately 30% of patients present with atypical symptoms [11]. Complications such as small bowel obstruction due to adhesion may develop after appendectomy and may require re-operation [1]. Delay in treatment may cause appendix perforation. Perforation causes an increase in morbidity and mortality. Therefore, achieving the correct diagnosis aims to avoid negative appendectomy and to reduce the risk of perforation [2]. In recent years, radiological imaging methods have been used frequently in diagnosis to improve diagnostic accuracy in suspected appendicitis cases. Studies have shown that computed tomography (CT) imaging has a sensitivity and specificity greater than 95% in the diagnosis of AA [12,13]. JB Puylaert used ultrasound (USG) with compression in 1986 to diagnose AA and it has been used ever since in the diagnosis [14]. Magnetic resonance imaging (MRI) has been preferred in the diagnosis because the diagnostic accuracy of USG is not satisfactory and varies according to the person, and CT, although having high diagnostic accuracy, exposes the patient to radiation. Increased appendix diameter has also been taken into consideration in imaging methods used in the diagnosis [15].

The complete blood count (CBC) is one of the most commonly used laboratory tests for the diagnosis of AA. Many studies have focused on the role of white blood cell (WBC), neutrophil-to-lymphocyte ratio (NLR), platelet distribution width (PDW), mean platelet volume (MPV), red cell distribution width (RDW), platelet count (PLT), lymphocyte (L), neutrophil (N), C-reactive protein (CRP), and Lymphocyte-C-reactive protein ratio (LCR) values in the diagnosis of AA [4,8,16,17,18]. We aimed to investigate the diagnostic efficacy of CBC parameters, CRP, and LCR in AA diagnosis and their relationship with appendix diameter.

Several studies have shown that NLR supports the diagnosis of acute appendicitis. In addition, an appendix diameter over 6 mm was found to be of significance in the diagnosis of AA. Evaluating these two together can strengthen the correct diagnosis of AA.

## 2. Material and Method

### 2.1. Study Place and Design

We retrospectively examined the patients who underwent appendectomy between 1 January 2012 and 30 June 2019 in the General Surgery Clinic of Gaziosmanpasa University, Faculty of Medicine. For this purpose, we recorded their age and gender using the hospital information management system database. We investigated the CBC parameters and CRP values at the time of admission to the emergency department. We reviewed the reports of the imaging methods used for the diagnosis. We recorded the appendix diameter in the USG and Abdominal CT reports. Measurements > 6 mm were accepted as pathological. According to the pathology results, we divided the results into two groups: LH and AA.

### 2.2. Exclusion Criteria

Among all appendectomy patients, those who did not undergo imaging and those whose pathology results indicated tumor were excluded from the study.

### 2.3. Statistical Methods

Statistical analysis of the data obtained from the study was performed using SPSS (Version 22.0, SPSS Inc., Chicago, IL, USA). Descriptive statistics were presented with mean ± standard deviation and median (min-max) according to data distribution for continuous variables. Descriptive statistics of categorical data were reported as numbers and percentages (%). The distribution of normality of data for statistical test selection was evaluated by the Shapiro–Wilk test. Comparisons of two independent groups were performed by the Mann–Whitney U test since the data were not distributed normally. The relationships between continuous variables were investigated with Spearman’s correlation coefficient in accordance with the data distribution. A chi-square test was used to investigate the relationship between categorical variables and to compare proportions.

Receiver operating characteristic (ROC) analysis was used to determine whether CBC parameters could be diagnostic and prognostic markers in the diagnosis of appendicitis. The area under the ROC curve (AUC) and 95% confidence intervals of this area were calculated. AUC was evaluated as 0.9–1: Excellent, 0.8–0.9: Good, 0.7–0.8: Fair, 0.6–0.7: Poor and 0.5–0.6: Fail. After the ROC analysis, the Youden index (maximum sensitivity and specificity) was used to determine the best cut-off point for the parameters found to be significant in ROC analysis. The success of cut-off points was evaluated with sensitivity, specificity, positive-negative predictive values, and likelihood ratio (+) values. The level of statistical significance was accepted as *p* < 0.05.

### 2.4. Ethics Committee Approval

This study was approved by the Local Clinical Research Ethics Committee of the Faculty of Medicine, Tokat Gaziosmanpaşa University, and was registered under number 19-KAEK-263.

## 3. Results

The study included 224 (59.7%) female and 151 (40.3%) male patients, 375 in total. Their mean age was 33.67 ± 13.29 (min–max: 15–89). The mean age of the women and men was statistically similar: 32.86 ± 13.68 (min–max: 15–89) and 34.87 ± 12.65 (min–max: 17–74) respectively (*p* = 0.153). We excluded 39 patients from the study because radiological imaging was not performed. Two patients of those who had the imaging were also excluded because of the presence of a pathological tumor.

**Figure 1:**
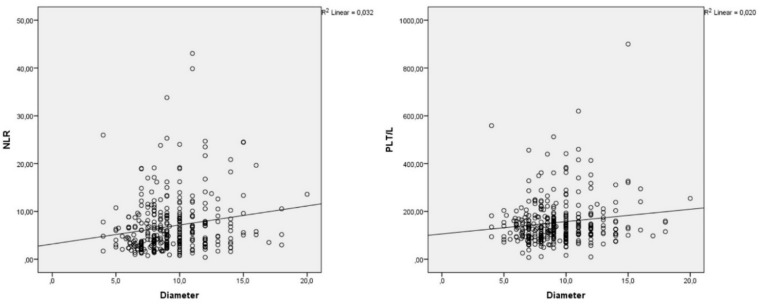
Scatter plot between the diameter of appendix and the NLR and PLT/L parameters.

One hundred and seventy-two (51.2%) of the diameter measurements were performed by CT and 164 (48.8%) by USG. The results of the correlation analysis between appendix diameter size and CRP and LCR are presented in Table 1. A statistically significant weak correlation was found between appendix diameter and WBC, Lymphocyte, Neutrophil, RDW, NLR, and PLT/L (Table 1). No correlation was found between the other CBC parameters (Table 1). The appendix diameter and the scatter plot between NLR and PLT/L are shown in Figure 1. 

As the appendix diameter increased, there was a parallel increase in WBC, N, RDW, NLR, PLT / L ratio, and a decrease in L count.

The comparison of the pathology results in the groups formed according to the appendix diameter is given in Table 2. The distribution of LH and appendicitis rates were significantly different between the groups (*p* < 0.001; Table 2). According to the pathology results, 88.4% of the patients with an appendix diameter of > 6 mm had appendicitis and 11.6% had LH. As for those with an appendix diameter of < 6 mm, 58.3% of them had appendicitis and 41.7% had LH.

The comparison of the CBC parameters in the groups is shown in Table 3. WBC, neutrophil, MPV, PDW, NLR, and diameter lengths were statistically different between the groups (Table 3). In the appendicitis group, WBC, neutrophil, MPV, and NLR were significantly higher and PDW was lower than the LH group. Other parameters were similar for PLT, Lymphocyte, RDW, CRP, and LCR (*p* > 0.05, Table 3).

We compared the CBC parameters in the patients with appendicitis and LH only among the patients with an appendix diameter of ≤6 mm: only MPV and RDW were statistically significantly different (*p* = 0.007, *p* = 0.006, respectively). In the appendicitis group, MPV was higher than that of the LH group (appendicitis: 9.17 ± 1.44; LH: 7.65 ± 1.03), whereas RDW was lower (appendicitis: 12.47 ± 2.03; LH: 15.15 ± 2.36). Other parameters; WBC, PLT, Lymphocyte, Neutrophil, PDW, CRP, NLR, LCR, and PLT/L were statistically similar in the pathology groups (respectively, *p* = 0.841, *p* = 0.437, *p* = 0.752, *p* = 0.841, *p* = 0.403, *p* = 0.388, *p* = 0.931, *p* = 0.388, *p* = 0.886).

**Figure 2:**
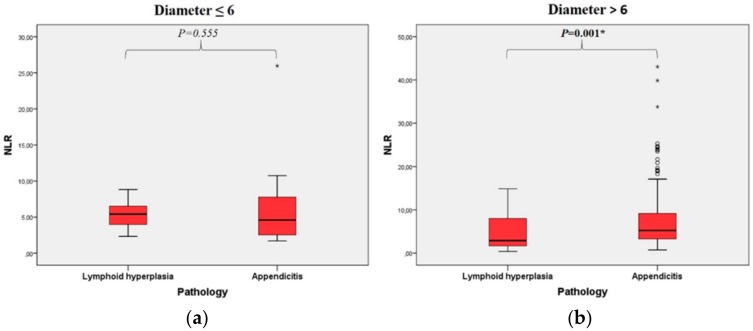
Box plot of neutrophil-to-lymphocyte in the group < 6 in diameter (**a**) and in the group > 6 in diameter (**b**) according to pathology groups.

WBC, neutrophil, PDW, and NLR values were significantly different between appendicitis and hyperplasia groups in patients with an appendix diameter of >6 mm (Table 4). NLR distributions in the pathology groups according to the diameter groups are shown in Figure 2 separately. There was no difference in PLT, Lymphocyte, MPV, RDW, CRP, LCR, and PLT/L (Table 4).

**Figure 3:**
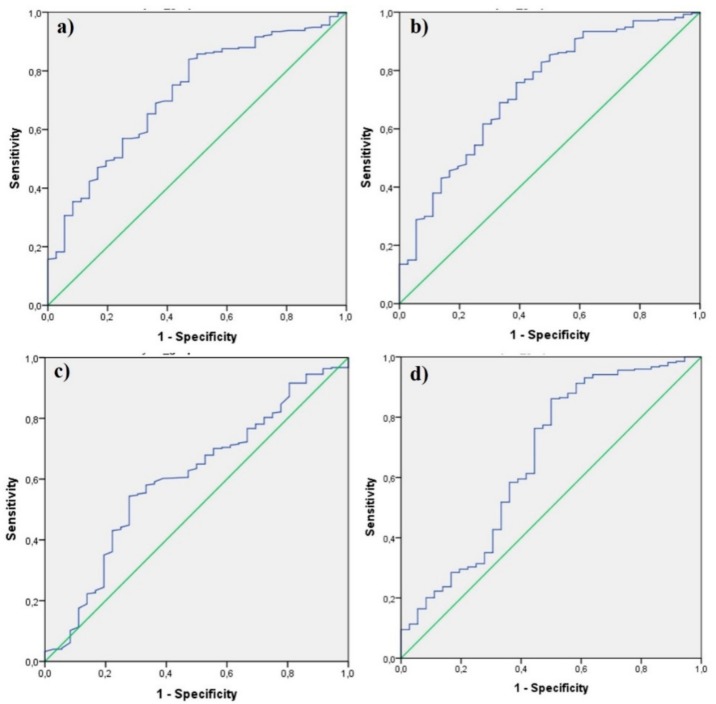
ROC curves of white blood cell (**a**), neutrophil-to-lymphocyte ratio (**b**), platelet distribution width (**c**), and neutrophil (**d**).

A ROC analysis to determine whether CBC parameters can be a diagnostic and prognostic marker in patients with an appendix diameter of >6 mm revealed PLT (AUC: 515), Lymphocyte (AUC: 513), MPV (AUC: 570), RDW (AUC: 531), CRP (AUC: 551), LCR (AUC: 550) and PLT (AUC: 521) meaningless (*p* > 0.05). The area under the curve was found to be statistically significant for WBC, Neutrophil, PDW, and NLR (Table 5, Figure 3). 

The sensitivity of the NLR score (cutoff = 2.6057) in the diagnosis of appendicitis was 86.1% and the selectivity was 50% in patients with an appendix diameter > 6 mm (Table 5). ROC results of WBC, Neutrophil, and PDW parameters are presented in Table 5.

## 4. Discussion

In this study, WBC, Neutrophil, MPV and NLR values were found to be significantly higher in patients with acute appendicitis confirmed by the pathology report than those who did not have a compatible pathology report for appendicitis. In addition, PDW values were significantly higher in the negative appendectomy group. In the radiological imaging (USG and CT) for the purpose of diagnosing acute appendicitis, the positive acute appendicitis rate was 88.4% in patients with an appendix diameter above 6 mm and was statistically significant compared to the group below 6 mm. We found that C-reactive protein (CRP) and lymphocyte CRP ratio (LCR) were not useful in predicting acute appendicitis.

We found that increased WBC count, Neutrophil count, MPV and NLR value may help in diagnosing acute appendicitis. PDW was found to be lower in patients with acute appendicitis. In addition, when we evaluated the diagnosis of acute appendicitis with the appendix diameter, we found that the diameter provides beneficial in making the diagnosis of AA. Our results confirm that evaluating complete blood count parameters, as shown in many studies, can help in the diagnosis of AA. WBC count and neutrophil count are the earliest indicators of inflammation in acute appendicitis. However, they are not a specific marker and are also commonly elevated in patients with other inflammatory conditions, thus this should be kept in mind for the differential diagnosis [19]. MPV and PDW are associated with the function and activation of platelets [20]. When MPV and PDW used for the diagnosis of acute appendicitis, different results were obtained. In some studies, it was stated that these values increase, while others decrease or do not differ [21,22,23,24]. In our study, we found that MPV value increased and PDW value decreased in patients with AA. In patients with an appendix diameter > 6 mm, PDW remained significant and MPV lost its significance. Interestingly, we only found differences in MPV and RDW parameters in the pathology results of patients with an appendix diameter of ≤6 mm. MPV was higher and RDW was lower in patients with appendicitis.

The diagnosis of acute appendicitis is mainly made by evaluating the patient’s symptoms, history, and findings of physical examination. In patients with suspected AA, the aim is to perform the necessary surgical treatment without a delay and also to avoid unnecessary appendectomy. For this purpose, imaging methods are frequently used in suspicious patients. By means of CT and USG, the radiologist looks for any possible positive findings such as: appendix diameter increase, appendix wall thickening, periapendicular fatty tissue heterogenecity, appendicolitis. An appendix diameter over 6 mm has been found to be significant in many studies [25,26,27]. In our study, we found that although appendix diameter increase was significant, 11.6% had negative appendectomy. When combined with appendix diameter we found WBC, neutrophils, PDW and NLR to be significant parameters for increasing the diagnostic accuracy of AA and for avoiding negative appendectomy. We found that NLR has the highest sensitivity in this patient group with 86%. Although the sensitivity was high, the specificity was 50% for NLR. PDW was found to have the highest specificity rate of 72.2%. These biomarkers alone usually have high sensitivity and low specificity. The combination of these biomarkers can increase specificity. Goodman et al. reported that NLR is a more useful parameter in predicting acute appendicitis than the total number of leukocytes [28]. NLR is a useful, simple and inexpensive marker of inflammation, which is easily calculated from a complete blood count [8]. In many studies, NLR has been shown to be a good biomarker in inflammatory conditions such as acute cholecystitis, diverticulitis and inflammatory bowel disease, as well as a prognostic indicator of malignancies [29,30,31,32].

In our study, we found that CRP and LCR do not contribute to diagnosis. The reason for this may be that CRP begins to rise 8–12 h after the onset of inflammation and reaches its peak in 24–48 h. However, in some studies, it has been used and found effective in CRP scoring systems [33,34]. In another study, it was found that CRP supports the clinical diagnosis of acute appendicitis in patients with typical clinical features [35].

There are some limitations of our study’s being retrospective in nature. Patients who attended the emergency department with suspicion of acute appendicitis but were not operated on were not included in the study. A comparison of complete blood count and biochemical parameters between nonoperated patients and operated patients and an evaluation of imaging findings in these patients could not be made. Findings in patients who have not been operated could be evaluated in further prospective studies. Comparing the diagnostic accuracy with blood tests performed on patients with suspected appendicitis measured by USG and CT is the strength of our study. The originality of this study lies in the fact that patients with suspected appendicitis, even after USG and CT evaluation, can benefit from a further CBC parameters interpretation. In patients with suspected clinical symptoms of acute appendicitis and an appendix diameter greater than 6 mm, a further evaluation of complete blood count parameters may strengthen the diagnostic value. Complete blood count is very useful basic laboratory test that is of low cost. NLR has high sensitivity and also has a positive predictive value as high as 92%. 

## 5. Conclusions

In patients with suspected acute appendicitis, elevated Neutrophil, MPV and NLR and decreased PDW are useful biomarkers for the diagnosis of AA. Appendix diameter measurements with USG and CT have 88.4% diagnostic accuracy. Assessment of appendix diameter and complete blood count parameters can be used together to increase the diagnostic value of AA.

## Figures and Tables

**Table 1:** Correlation analysis results between appendix diameter and CBC parameters.

CBC Parameters		WBC	PLT	L	N	MPV	PDW	RDW	CRP	NLR	LCR	PLT/L
Diameter	*r*	0.159 **		−0.144 **	0.154 **			0.133 *		0.195 **		0.114 *
	*p*	0.004	0.270	0.008	0.005	0.321	0.094	0.015	0.267	< 0.001	0.074	0.037
	*n*	334	334	334	334	334	334	334	257	334	257	334

Spearman’s rho correlation coefficient statistically significant * *p* < 0.05, ** *p* < 0.01.WBC: white blood cell; NLR: neutrophil-to-lymphocyte ratio; PDW: platelet distribution width; MPV: mean platelet volume; RDW: red cell distribution width; CRP: C-reactive protein; PLT: platelet count; LCR: lymphocyte-C-reactive protein ratio, L: lymphocyte; N: neutrophil.

**Table 2:** Comparison of pathology results in groups divided by appendix diameter.

Diameter		Pathology		Total	*p* Value
		−	+		
Diameter	Normal (≤6 mm)	10	14	24	<0.001 *
		41.7%	58.3%	100%	
	High (>6 mm))	36	274	310	
		11.6%	88.4%	100%	
Total		46	288	334	
		13.8%	86.2%	100%	

* Chi square test statistically significant.

**Table 3:** Comparison of CBC parameters in groups divided by appendix diameter.

CBC Parameters	Pathology Groups	N	Mean	SD	Median	Min	Max	*p* Values
WBC	LH	46	10.25	3.11	10.33	3.47	16.49	<0.001 **
	A	288	13.19	6.01	12.79	3	88.4	
PLT	LH	46	229.0	58.0	221.5	110	358	0.569
	A	288	235.3	61.9	227.5	80	485	
Lymphocyte	LH	46	2.28	3.15	1.81	0.7	22.71	0.899
	A	288	2.02	1.87	1.78	0.4	21.97	
Neutrophil	LH	46	7.24	3.28	6.86	1.79	14.39	<0.001 **
	A	288	10.55	6.77	9.98	1.59	78.37	
MPV	LH	46	8.53	1.58	8.16	6.14	12.6	0.011 *
	A	288	9.15	1.72	9.33	3.39	20.1	
PDW	LH	46	16.81	3.70	17.61	9.1	21.65	0.038 *
	A	288	15.26	4.21	15.6	1.1	23	
RDW	LH	46	13.68	1.98	13.30	11.03	20.04	0.059
	A	288	13.16	2.01	12.6	10.12	27.2	
CRP	LH	32	50.58	68.24	15.05	0.4	266	0.933
	A	225	46.11	63.23	19.3	0.23	331	
NLR	LH	46	4.85	3.57	4.01	0.4	14.84	0.003 **
	A	288	7.22	6.13	5.21	0.76	43.04	
LCR	LH	32	0.42	0.86	0.10	0	4.25	0.960
	A	225	0.47	1.38	0.09	0	12.3	
PLT/L	LH	46	139.0	63.3	131	9.48	362.8	0.649
	A	288	156.3	101.3	131.1	7.42	900	
DIAMETER	LH	46	7.89	2.16	7.5	4	13	<0.001 **
	A	288	9.53	2.63	9	4	20	

Mann–Whitney U test statistically significant * *p* < 0.05, ** *p* < 0.01, LH: lymphoid hyperplasia, A: Appendicitis, SD: standard deviation, WBC: white blood cell, NLR: neutrophil-to-lymphocyte ratio, PDW: platelet distribution width, MPV: mean platelet volume, RDW: red cell distribution width, CRP: C-reactive protein, PLT: platelet count, LCR: lymphocyte-C-reactive protein ratio, PLT/L: platelet count-lymphocyte ratio.

**Table 4:** Comparison of CBC parameters in patients with an appendix diameter > 6 mm.

CBC parameters	Pathology Groups	N	Mean	SD	Median	Min	Max	*p* Values
WBC	LH	36	10.01	3.16	8.85	3.47	16.49	<0.001 **
	A	274	13.27	6.07	12.79	3	88.4	
PLT	LH	36	231.7	60.3	221.5	110	358	0.772
	A	274	235.1	62.1	227.5	80	485	
Lymphocyte	LH	36	2.43	3.55	1.81	0.70	22.71	0.801
	A	274	2.04	1.92	1.78	0.40	21.97	
Neutrophil	LH	36	6.91	3.39	6.03	1.79	14.39	<0.001 **
	A	274	10.63	6.86	10	1.59	78.37	
MPV	LH	36	8.77	1.63	8.83	6.14	12.6	0.170
	A	274	9.15	1.73	9.33	3.39	20.1	
PDW	LH	36	16.71	3.95	17.56	9.1	21.65	0.048 *
	A	274	15.15	4.17	15.4	1.1	23	
RDW	LH	36	13.27	1.67	12.95	11.03	17.1	0.548
	A	274	13.19	2.01	12.7	10.12	27.2	
CRP	LH	26	44.64	72.3	11.85	0.4	266	0.391
	A	216	45.81	63.7	18.85	0.23	331	
NLR	LH	46	13.68	1.98	13.3	11.03	20.04	0.001 **
	A	288	13.16	2.01	12.6	10.12	27.2	
LCR	LH	26	0.489	0.94	0.17	0	4.25	0.407
	A	216	0.486	1.41	0.09	0	12.3	
PLT/L	LH	36	139.7	67.5	129.4	9.48	362.86	0.684
	A	274	155.9	100.6	130.6	7.42	900	

Mann–Whitney U test statistically significant * *p* < 0.05, ** *p* < 0.01; LH: lymphoid hyperplasia, A: appendicitis, SD: standard deviation; WBC: white blood cell, NLR: neutrophil-to-lymphocyte ratio, PDW: platelet distribution width, MPV: mean platelet volume, RDW: red cell distribution width, CRP: C-reactive protein, PLT: platelet count, LCR: lymphocyte-C-reactive protein ratio, PLT/L: platelet count-lymphocyte ratio.

**Table 5:** ROC curve results for CBC parameters in the group with a diameter > 6.

CBC Parameters	Cut Off	AUC (95% CI)	*P*	Sensitivity	Specificity	PPV	NPV	L +
WBC	9.065	0.720 (0.635–0.805)	<0.001	83.9%	52.8%	93.1%	30.1%	1.78
N	7.485	0.734 (0.645–0.823)	<0.001	75.9%	61.1%	93.7%	25%	1.95
PDW	15.75	0.602 (0.503–0.700)	0.048	54.4%	72.2%	93.7%	17.2%	1.96
NLR	2.605	0.663 (0.555–0.770)	0.001	86.1%	50%	92.9%	32.1%	1.72

AUC: area under curve, CI: confidence Interval, PPV: positive predictive value; NPV: negative predictive value, L: likelihood ratio; WBC: white blood cell, NLR: neutrophil-to-lymphocyte ratio, PDW: platelet distribution width, N: neutrophil.
